# Successful delivery after vaginal occlusion in addition to cerclage in a trachelectomy patient with recurrent second trimester pregnancy loss: a case report

**DOI:** 10.1002/ccr3.84

**Published:** 2014-06-03

**Authors:** Kirstine Sneider, Mette Østergaard Poulsen, Christian Ottosen, Jens Langhoff-Roos

**Affiliations:** 1 Center of Clinical Research, Vendsyssel Hospital, Aalborg UniversityAalborg, Denmark; 2 Department of Clinical Medicine, Aalborg UniversityAalborg, Denmark; 3 Department of Gynaecology, Rigshospitalet, University of CopenhagenCopenhagen, Denmark; 4 Department of Obstetrics, Rigshospitalet, University of CopenhagenCopenhagen, Denmark

**Keywords:** Abdominal cerclage, cervical amputation, second trimester pregnancy loss, trachelectomy, transvaginal cervical cerclage, vaginal closure, vaginal occlusion

## Abstract

**Key Clinical message:**

Pregnancy outcome after trachelectomy has high risk of complications such as second trimester pregnancy loss and preterm birth. We report beneficial effect of a simple procedure of vaginal occlusion in addition to cerclage in a patient with trachelectomy and two prior second trimester pregnancy losses.

## Introduction

Trachelectomy is a fertility preserving treatment in women with early-stage uterine cervical cancers or recurrent severe dysplasia. The operation involves surgical amputation of the uterine cervix and placement of a suture in the cervical canal (intracervical cerclage). Pregnancy after trachelectomy is associated with second trimester loss and extreme preterm birth. According to a review by Jolley et al. [1], the rate of second trimester pregnancy losses before 24 weeks was 19/200 (9.5%) of women who became pregnant after trachelectomy. The rate of preterm delivery between 24 and 34 weeks was 27/200 (13.5%).

According to Danish National Guidelines, patients with recurrent second trimester losses or extremely preterm delivery and shortening of the cervix should be offered prophylactic cerclage before or early in the following pregnancy. In women with previous failed transvaginal cervical cerclage or major cervical surgery such as trachelectomy, an abdominal cerclage is indicated. The guideline is based on review of literature with emphasis on reviews by Berghella et al. [2] and Burger et al. [3].

There is a paucity of reported alternative solutions when cerclage fails. In 1981, Saling [4] described a surgical procedure, total cervical occlusion, as an alternative to cerclage in preventing recurrent second trimester pregnancy losses. The technique involves denuding the cervical mucosa before stitching to completely occlude the cervical os and potentially reduce the risk of ascending infections.

## Case Presentation

A 32-year-old gravida 3 para 0 induced first trimester abortions 3, presented with recurrent dysplasia. As she previously underwent five conisations, it was decided to perform a simple trachelectomy. Fertility counseling was given before the operation. The operation involved surgical amputation of the uterine cervix just below the isthmus. After the trachelectomy, a suture was placed around the cervical canal (intracervical cerclage) establishing a ∼5-mm neocervix. The patient conceived 6 months later after insemination. Reactive stenosis necessitated dilation of the cervical canal prior to insemination.

Her first pregnancy after trachelectomy ended in preterm prelabour rupture of membranes (PPROM) in gestational week 15. Dilatation and curettage of the uterus was performed without removing the sutures. In her second pregnancy after trachelectomy, an abdominal cerclage (Mersilene; Ethicon, Birkerød, Denmark) was placed in week 9. The procedure was complicated by an immobile uterine body caused by adhesions to the residual cervix. It was possible, however, to obtain correct placement and tightening of the Mersilene band at cervicouterine transition. Vaginal examination showed no dilatation of the external cervical os before the operation. Unfortunately, in week 16, she had recurrent PPROM and due to the presence of abdominal cerclage a minor cesarean section (sectio parva) was performed. In both pregnancies, the fetuses were normally developed.

As she suffered recurrent stenosis, dilatation of the cervix was repeated before insemination. In her third pregnancy after trachelectomy, she was offered a procedure of vaginal occlusion and accepted. After a normal sonography in week 13, the upper part of the vagina was closed transversely just below the cervical os with two layers of resorbable continuous sutures (Monocryl; Ethicon). Prior to the closure, the upper vaginal wall was de-epithelialized 1 cm below the external os, thus facilitating adhesion of the vaginal walls. The intracervical and abdominal cerclages remained in situ. The patient was discharged from the hospital the same day, and had vaginal progesterone 200 mg daily until week 34. The pregnancy progressed without complications except for a few episodes of spotting. We did not prescribe activity restriction to the woman, but she chose bed rest for several months after surgery. At 37+0 weeks, she gave birth to a healthy male infant via an elective caesarean section. At the caesarean delivery, the closure of the vagina was bluntly dilated passing a Hegar 8 from inside of the uterus through the cervical canal and into the vagina. The abdominal cerclage was unchanged in an appropriate position.

Subsequent examination of the vagina 2 months after delivery showed no signs of stenosis and only minimal scarring. The patient reported transient pain only during the first sexual intercourse after delivery and subsequently no dyspareunia.

The patient has remained without evidence of recurrence of cervical dysplasia.

## Discussion

Trachelectomy is usually followed by a concomitant cerclage. It is unclear whether abdominal cerclage is preferable to transvaginal cerclage. Transabdominal cerclage is more invasive as it requires laparotomy/laparoscopy and requires delivery by cesarean section. For this reason, most researchers do not advocate it as a routine but reserve the procedure to those cases with failure of transvaginal cerclage. In this case, an abdominal cerclage was placed at 9 weeks in a woman with a history of PPROM and second trimester pregnancy loss. Unfortunately, this treatment was unsuccessful as the patient suffered PPROM in week 16. In this case, the cervix was very short and mutilated and the intra-abdominal part appeared adherent to the surroundings. Takada et al. [5] report a similar case of failed abdominal cerclage in a trachelectomy patient. They emphasize the difficulties in performing a sufficient closure of the residual cervix in trachelectomy patients as the cerclage has to be placed in the higher parts of the uterus where the muscle layer is thicker.

The reported use of vaginal occlusion in trachelectomy patients is limited. Mathevet et al. [6] report beneficial effects of the procedure in decreasing the rate of second trimester loss 20% to 10% among 65 pregnancies in patients treated with trachelectomy. Based on their review of the literature, however, Jolley et al. [1] do not advocate the routine of vaginal occlusion but reserve its use to only those patients with complications of cerclage or with a history of pregnancy loss. In our patient, it is noticeable that the vaginal closure is easily opened from inside the uterus, while the abdominal cerclage is still in place. Thus, the vaginal occlusion functions more as a protecting membrane than a mechanical support. The cerclage tightens the cervical canal and secures a cervical plug, and the vaginal occlusion prevents ascension of microorganisms from the vagina to the cervix. In this case, the combination of cerclage and vaginal occlusion resulted in an uncomplicated pregnancy carried until term. Technically, it is quite easy to replicate this intervention; it is not more demanding than the application of a vaginal cerclage.

## Conclusion

Successful pregnancy outcome is possible after trachelectomy. In this case, vaginal occlusion in addition to transvaginal intracervical and abdominal cerclage was a safe procedure with minimal complications. The method might be considered in trachelectomy patients with a history of second trimester loss or preterm delivery.
